# Clinico-epidemiological profile findings of the screened population under NPCDCS–Ayush (Integration of Homeopathy along with Yoga): a pilot project

**DOI:** 10.3389/fpubh.2026.1793635

**Published:** 2026-05-21

**Authors:** Roja Varanasi, Shashi Giri, Khushboo Garg, Amit Srivastava, Gurudev Choubey, Jogendra Singh Arya, Ramesh Bawaskar, Umakanta Prusty, Ch. Raveender, Chetna Deep Lamba, Anupriya Chaudhary, Jyotika Bhatti, Manjula Narang, Arvind Kumar, Praveen Oberai, Anil Khurana, Raj K. Manchanda, Leimapokhpam Swasticharan, NPCDCS-AYUSH integration project study group

**Affiliations:** 1 Central Council for Research in Homeopathy, New Delhi, India; 2 Central Research Institute (Homeopathy), Lucknow, India; 3 Regional Research Institute (Homeopathy), Siliguri, India; 4 Regional Research Institute (Homeopathy), Mumbai, India; 5 Regional Research Institute (Homeopathy), Puri, India; 6 Regional Research Institute (Homeopathy), Gudivada, India; 7 National Commission of Homeopathy, New Delhi, India; 8 Nehru Homoeopathic Medical College and Hospital, New Delhi, India; 9 Ministry of Health and Family Welfare, New Delhi, India

**Keywords:** Ayush integration, clinico-epidemiological profile, Homeopathy, India, non-communicable diseases, NPCDCS, opportunistic screening

## Abstract

**Background:**

Non-communicable diseases (NCDs) are a major global health challenge, accounting for nearly 74% of deaths worldwide, with the highest burden in low- and middle-income countries. In India, 66% of total deaths are attributed to NCDs, reflecting a rapid epidemiological transition and a dual burden of disease. To address this, the Ministry of Health and Family Welfare launched the National Programme for Prevention and Control of Cancer, Diabetes, Cardiovascular Diseases and Stroke (NPCDCS) in 2010, integrating Ayush systems (Homeopathy along with Yoga) to strengthen prevention and early management in 2015.

**Objectives:**

To determine the presence and distribution of NCDs through opportunistic screening and to describe their clinico-epidemiological profile and correlates under the NPCDCS–Ayush Integration Project.

**Methods:**

A cross-sectional opportunistic screening was conducted from 2015 to 2021 across four districts of India under the NPCDCS–Ayush integration initiative implemented by the Central Council for Research in Homeopathy. Standardized NPCDCS screening tools were used to collect sociodemographic, clinical, and behavioral data. Descriptive and inferential analyses were performed to assess NCD prevalence and associated risk factors.

**Results:**

A considerable proportion of the screened population (55.3%) was found to be affected by one or more NCDs, particularly hypertension and diabetes mellitus. The most common diagnoses were HTN (39.2%), high-normal/pre-HTN (20.9%), pre-diabetes (9.4%), and diabetes (5.4%). A higher burden of NCDs was observed among middle-aged and elderly individuals, with significant associations noted with physical inactivity, tobacco use, and unhealthy dietary habits. The integration of Ayush services enhanced community participation and facilitated early detection of NCDs, highlighting their potential to strengthen population-based screening and preventive strategies.

**Conclusion:**

The study highlights the growing burden of NCDs and demonstrates that Ayush-integrated opportunistic screening is a feasible approach for early detection within India’s national NCD control framework. Integration of Ayush services may improve community reach and acceptability, facilitating timely identification of both disease and pre-disease states, and highlighting the need to strengthen community-based screening and targeted health promotion, to reduce long-term NCD burden.

## 1 Introduction

Non-communicable diseases (NCDs) account for almost 74% of all deaths worldwide, with around 17 million people dying annually between the age groups of 30 and 69 years. A majority (86%) of these deaths are reported from low and middle-income countries (1). In India, 66% of all deaths are attributed to NCDs, with a 22% probability of premature deaths (2). Of all the NCDs, cardiovascular diseases are the leading cause of mortality, followed by cancer. India is a developing nation in South Asia, witnessing fast economic growth and a shift in disease burden from communicable diseases to NCDs (3–8). Thus, current epidemiological transition can be considered as one with high morbidity and a double burden of disease (4, 6, 9).

People of all age groups throughout the world are vulnerable to developing NCDs when exposed to risk factors such as unhealthy diets, physical inactivity, tobacco smoke, alcohol or air pollution (1). NCDs have an adverse economic impact, resulting in increased health expenditure and loss of income, forcing families into poverty (10, 11). Early detection of NCDs may help with risk stratification, early referral, and enhance the efficiency of NCD management within the NPCDCS framework.

The National Programme for Prevention and Control of Diabetes, Cardiovascular Diseases and Stroke (NPCDCS) was launched by the Ministry of Health and Family Welfare (MoHFW), Government of India, in the year 2010 (12). Later, the National NCD monitoring framework and action plan were adopted to reduce premature deaths from NCDs by one-third by the year 2030 (13). Integration of Ayush was one of its mandates. Screening for risk factors and implementing strategies to modify them and mitigate the risk posed by NCDs is a challenging task that requires an innovative, community-friendly approach. The integrated approach brought together various agencies under the government in identifying and managing the NCDs. The Central Council for Research in Homeopathy (CCRH) implemented the NPCDCS-Ayush integration project (Homeopathy along with Yoga) in collaboration with the MoHFW in four districts of the country from 2015 to 2021. The objectives of this integration project were to promote health through behavior change, prevent NCDs through early diagnosis, reduce the NCD burden and risk factors, and manage them early through homeopathic medicinal treatment along with yoga as an add-on to standard care or as a standalone. This paper aims to present an overview of the clinico-epidemiological characteristics of the population screened under the Integrated NPCDCS Pilot Project (Homeopathy along with Yoga). It estimates the prevalence of modifiable and non-modifiable risk factors for major non-communicable diseases, analyses sociodemographic correlates among both healthy and morbid participants, and describes the demographic profile of the diagnosed population.

## 2 Methodology

### 2.1 Study design and setting

**Figure 1:**
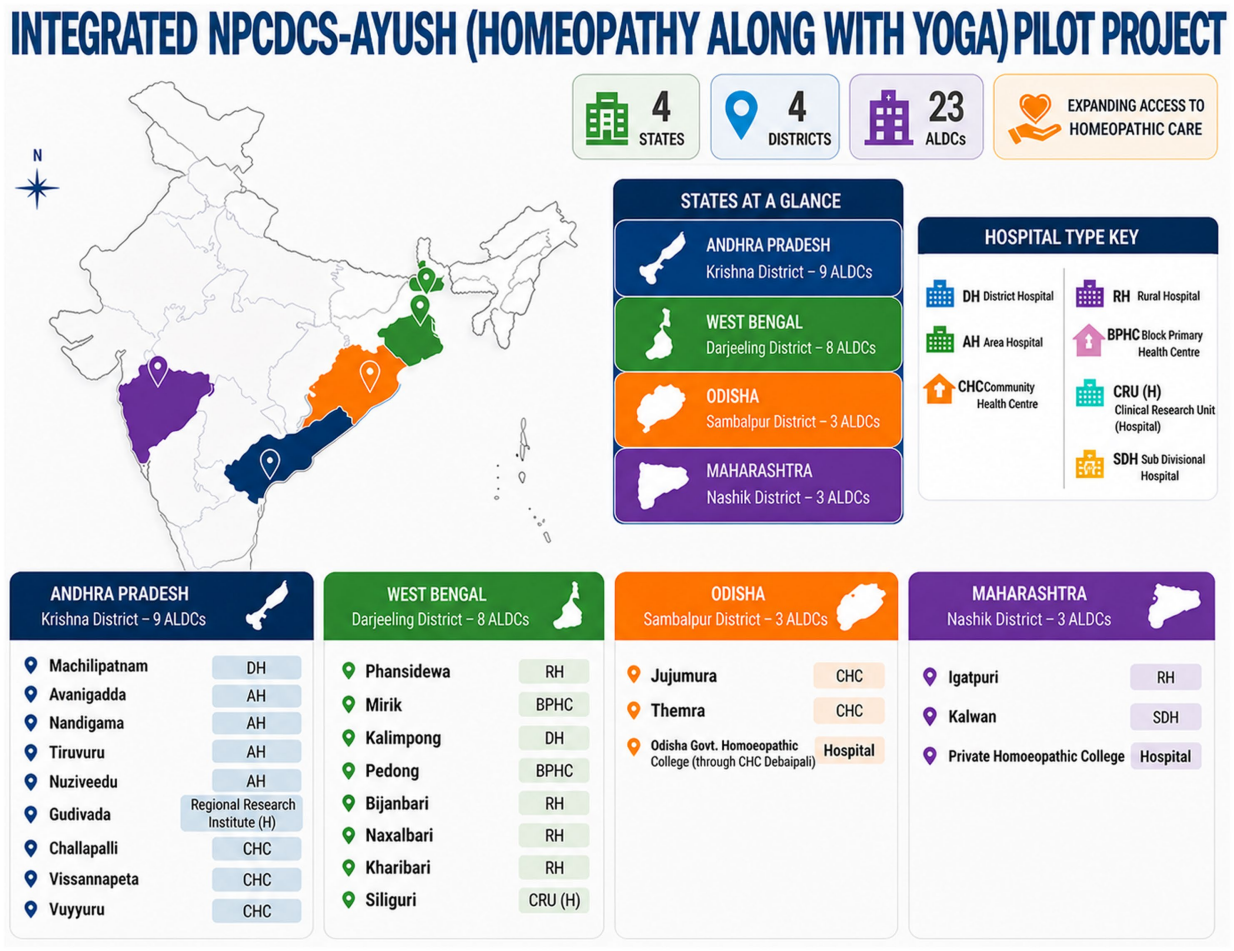
Geographical representation of the implementing districts of the NPCDCS-Ayush integration project.

**Figure 2:**
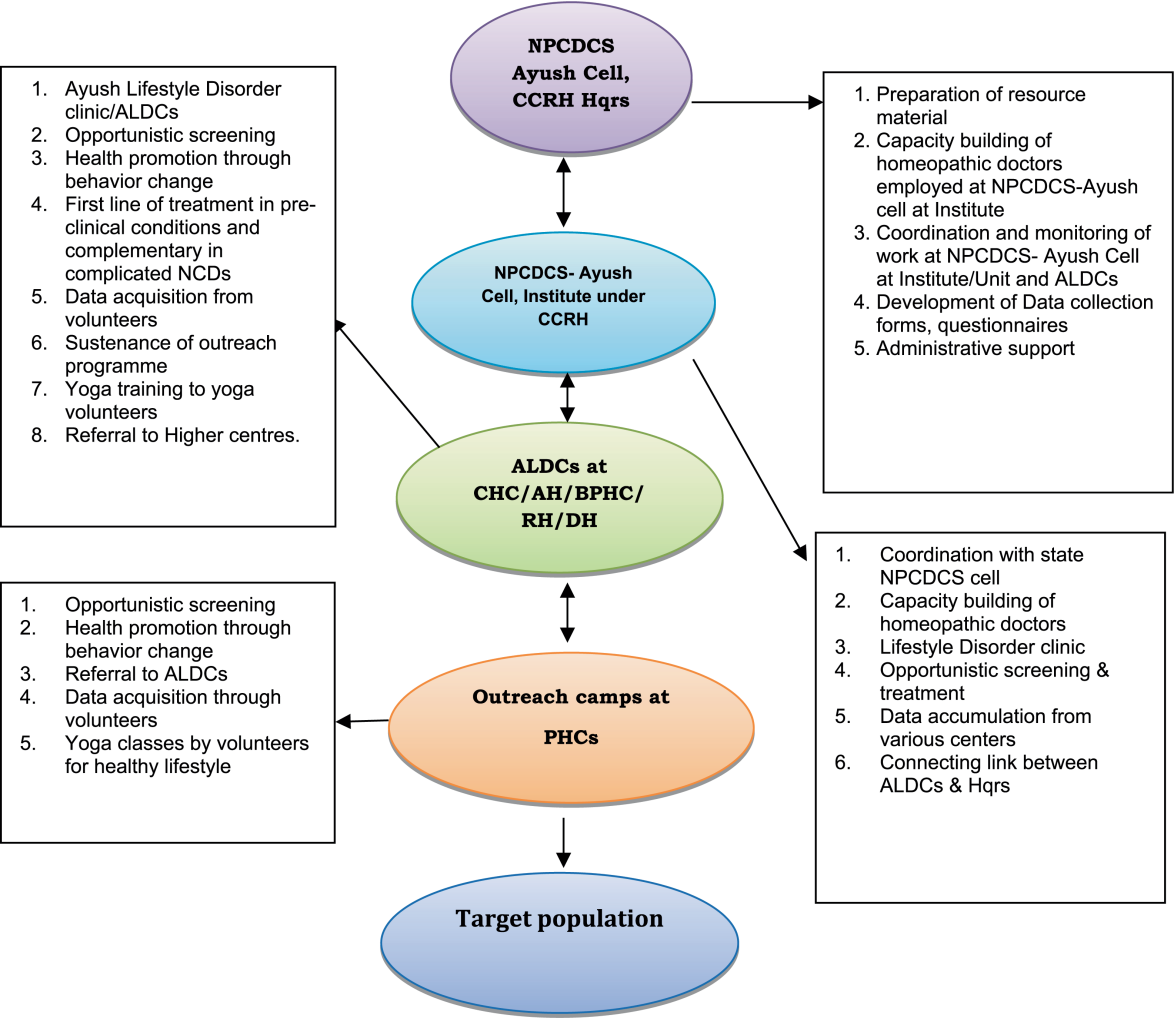
Roles and responsibilities at different levels of implementation.

This project was implemented in collaboration with the Directorate General of Health Services under the MoHFW, Government of India, in coordination with state health authorities. The study was conducted within the framework of the NPCDCS program. A multi-centric cross-sectional study design employing opportunistic screening was adopted across four purposively selected districts—Krishna (Andhra Pradesh), Darjeeling (West Bengal), Nashik (Maharashtra), and Sambalpur (Odisha). The study was carried out from September 2015 to April 2021 in Krishna and Darjeeling districts, while it was executed in Nashik and Sambalpur districts from May 2017 to May 2020. Ayush Lifestyle Disorder Clinics (ALDCs) were established at district hospitals, community health centers, and block-level primary health centers, based on feasibility assessments and site selection by the respective state health authorities, to support screening for pre-identified non-communicable diseases in tandem with the NPCDCS program of Govt. of India. Figure 1 outlines the geographical representation of the implementing districts of the NPCDCS-Ayush integration project. Twenty three ALDCs were set up at the state-identified District hospitals/Community Health Centers/Block Primary Health Centers across the four districts: nine ALDCs at Krishna, eight at Darjeeling, three each at Nashik, and Sambalpur. At the center, the pilot project was coordinated by the head of the organization and the central team at the headquarters of CCRH in New Delhi. At each identified district, regional project coordinators were identified, who were the regular officials of the implementing organization, i.e., CCRH. Under each regional project coordinator, a team of two institutionally qualified homeopathic physicians, one institutionally qualified Yoga instructor, one Data Entry Operator (DEO), and two Multi-Tasking Workers (MTW) were recruited for each ALDC. They were provided with two-day hands-on training before implementation of the project, and regular online and off-site monitoring was conducted to ensure proper deliberation of the assigned responsibilities. Administrative support for the ALDC’s functioning was also provided by the conventional medical heads at each integrated study site. Opportunistic screening was conducted through regular outreach camps and at established ALDCs with the engagement of community leaders. The study team, as mentioned above, was responsible for screening identified NCDs through outreach camps and at ALDCs, conducted health awareness sessions, and delivered educational lectures to promote behavioral changes. These included discouraging alcohol consumption, smoking, and tobacco use, while encouraging regular physical activity, adopting healthy dietary practices, and participating in yoga to promote preventive and promotive health at the community level. Figure 2 outlines the role and responsibilities at different levels of implementation.

### 2.2 Study participants

Participants of all genders and above 30 years of age visiting the outreach camps or ALDCs and giving verbal consent were eligible for participation in this survey, whereas children, adolescents and adults below 30 years and not providing verbal consent were excluded.

### 2.3 Data collection

The study adapted the Community-Based Assessment Checklist (CBAC) form for individuals above 30 years of age as per the operational guidelines of the NPCDCS program of the Government of India (14) and some of the recommendations of the World Health Organization (WHO) STEPwise approach to NCD risk factor surveillance (STEPS) (15).

Prior to the initiation of the study, the study staff underwent hands-on training for standardized data collection procedures through the Standard operating procedures. Instruments like weighing machine, BP instrument, a glucometer, measuring tape were provided to each ALDC for uniform data capturing across all four districts.

Participants visiting outreach camps at the community level or at ALDCs were screened for the presence of NCDs, and data were captured in structured formats according to the predefined standard operating procedures. Each participant’s demographic information and anthropometric measurements, weight, height, waist circumference, hip circumference, waist-to-hip ratio (WHR), and Body Mass Index (BMI), were recorded. Other information included family history related to NCDs, smoking and drinking habits, consumption of tobacco products, and exposure to occupational/environmental pollution. Physical activity was evaluated using the Indian Diabetes Risk Score (IDRS). The IDRS is a validated tool that includes physical activity as an important component for risk assessment. The physical activity was grouped into four categories according to participants’ usual occupational and daily activities: (i) vigorous exercise or strenuous work, (ii) moderate activity at work or home, (iii) mild activity at work or home, and (iv) no exercise with a sedentary lifestyle. These categories were assigned scores of 0, 10, 20, and 30, respectively, in line with the standard IDRS scoring system, where higher scores indicate lower levels of physical activity (16).

For analysis, individuals classified under “no exercise and sedentary at work/home” were considered physically inactive. This definition was used to estimate the prevalence of physical inactivity in the study population. Participants’ weight and height were measured in light clothing and without shoes. BMI was calculated as weight in kilograms/height in meters squared. Waist circumference and hip circumference were measured using constant tension tape. Waist circumference was measured at the end of a normal expiration, with arms relaxed at the sides, at the midpoint between the lower part of the lowest rib and the highest point of the hip on the mid-axillary line. Hip circumference was measured at the maximum curvature of the buttocks.

Apart from demographic profile and anthropometric measurements, physiological measurements included measurements of blood pressure and random blood sugar. Blood pressure was measured using calibrated equipment. It was measured three times, with an interval of at least 3 min between each measurement, and the average of the readings was reported according to the standard guidelines (17) Random blood glucose (RBG) was measured using a validated and calibrated glucometer. Pre-hypertension and pre-diabetes were classified as NCDs as they represent the early stages of disease rather than the normal states. They are characterized by quantifiable physiological abnormalities (such as poor glucose regulation and raised blood pressure), early organ damage, and a high likelihood of progressing to type 2 diabetes mellitus and hypertension (HTN). Therefore, the diseased group included individuals with diagnosed chronic diseases as well as those with early risk conditions such as pre-diabetes and pre-hypertension. For the classification of patients as hypertensive or non-hypertensive, Indian guidelines on hypertension-IV (IGH-IV) were followed. HTN was defined according to the *Indian Guidelines on Hypertension-IV (IGH-IV)* as systolic blood pressure (SBP) ≥140 mmHg and/or diastolic blood pressure (DBP) ≥90 mmHg, or a self-reported history of antihypertensive medication use. A SBP under 130 mmHg and a DBP under 85 mmHg is considered normal, while the prehypertension stage is reached when the SBP falls between 130 and 139 mmHg or the DBP falls between 85 and 89 mmHg. An SBP between 140 and 159 mmHg or a DBP between 90 and 99 mmHg indicates Stage 1 HTN; an SBP between 160 and 179 mmHg or a DBP between 100 and 109 indicates Stage 2 HTN, and an SBP of 180 mmHg or higher or a DBP of 110 mmHg or higher indicates Stage 3 HTN (18). RBG levels were used to classify patients as non-diabetic, pre-diabetic, and diabetic, based on criteria established by the WHO and the Indian Council of Medical Research (ICMR). Diabetes was defined using *WHO and ICMR criteria* as RBG ≥ 200 mg/dL (11.1 mmol/L), or a self-reported history of diabetes or use of antidiabetic medication. An RBG level less than 140 mg/dL is considered normal; between 140 and 200 mg/dL is prediabetes, and 200 mg/dL or higher indicates diabetes mellitus (DM) (19, 20). Obesity was assessed using BMI, with values ≥25 kg/m^2^ classified as overweight and ≥30 kg/m^2^ as obese, as per the WHO classification. Outreach camps served as the entry point, referring to high-risk individuals to ALDCs or higher healthcare facilities. The high-risk individuals screened at the ALDCs.

### 2.4 Sample size

This pilot project did not use a pre-determined sample size calculation. Instead, a purposive convenience sampling method was applied. A total of 197,229 participants were included in the study.

### 2.5 Statistical analysis

Data were recorded in pre-designed excel sheets, followed by data cleaning prior to analysis. Statistical analysis was performed using SPSS version 20. The distribution of sociodemographic profile and various risk factors was summarized using mean (standard deviation)/frequency (proportion) as per the variables. All estimates are presented with 95% confidence intervals (CIs).

Further analyses were conducted stratified by age group, gender, place of residence, occupation, and education. The clinico-epidemiological characteristics of the screened participants were assessed to examine associations between sociodemographic factors and disease/morbidity status using univariate and multivariate logistic regression models. Both crude odds ratios (ORs) and adjusted odds ratios (AORs) are reported. A *p*-value of <0.05 was considered statistically significant.

### 2.6 Ethical clearance

The study was conducted in accordance with the principles outlined in the Declaration of Helsinki. All the respondents gave written informed consent to participate in the study and were informed that their information would be kept anonymous and confidential. The pilot project was approved by the 24th Central Ethical Committee of CCRH, New Delhi (1-3/2019-20/CCRH/Tech./24th EC).

## 3 Results

A total of 197,229 individuals aged 30 years and above were screened under the integrated pilot project for any NCD at ALDCs and outreach camps, among whom 59.4% were females and 53.3% resided in urban areas. The majority (52.2%) of participants were aged between 30 and 49 years, 31.9% were overweight, and 16.7% obese, and nearly two-thirds (68.5%) were illiterate. Among all participants, 55.3% had one or more NCDs. A higher proportion of obesity was observed among urban residents (22.2%) compared to rural (10.5%) participants. Females showed a higher prevalence of overweight and obesity (50.9%) than males (45.6%) (Table 1).

**Table 1:** Demographic profile of study participants.

Variables		Total *N* = 197,229	Male *N* = 80,019 (40.6%)	Female *N* = 117,210 (59.4%)	Rural *N* = 92,084 (46.7%)	Urban *N* = 105,145 (53.3%)
		*n* (%)	*n* (%)	*n* (%)	*n* (%)	*n* (%)
Age (in years)	30–39	51,467 (26.1)	17,252 (21.6)	34,215 (29.2)	24,392 (26.5)	27,075 (25.8)
	40–49	51,533 (26.1)	19,585 (24.5)	31,948 (27.3)	23,572 (25.6)	27,961 (26.6)
	50–59	45,120 (22.9)	18,639 (23.3)	26,481 (22.6)	20,583 (22.4)	24,537 (23.3)
	60–69	33,554 (17.0)	15,586 (19.4)	17,968 (15.3)	15,958 (17.3)	17,596 (16.7)
	≥70	15,555 (7.9)	8,957 (11.2)	6,598 (5.6)	7,579 (8.2)	7,976 (7.6)
Education	Illiterate	134,962 (68.5)	47,622 (59.6)	87,340 (74.6)	63,012 (68.4)	71,950 (68.5)
	Junior High School	39,342 (20.0)	18,667 (23.4)	20,675 (17.7)	19,352 (21.0)	19,990 (19.0)
	Senior High School	8,989 (4.5)	4,963 (6.2)	4,026 (3.4)	4,411 (4.8)	4,578 (4.4)
	Undergraduate	13,770 (7.0)	8,689 (10.8)	5,081 (4.3)	5,307 (5.8)	8,463 (8.1)
Occupation	Homemaker	72,297 (36.7)	-	72,297 (61.7)	31,451 (34.2)	40,846 (38.9)
	Professional	12,732 (6.5)	9,168 (11.5)	3,564 (3.1)	5,938 (6.4)	6,794 (6.5)
	Manual laborer	62,007 (31.5)	34,972 (43.7)	27,035 (23.1)	30,808 (33.5)	31,199 (29.7)
	Others	50,031 (25.4)	35,803 (44.8)	14,228 (12.1)	23,887 (25.9)	26,144 (24.9)
BMI (kg/m^2^)*	Underweight (≤18.5)	14,233 (7.2)	5,449 (6.8)	8,784 (7.5)	9,088 (9.9)	5,145 (4.9)
	Normal (18.51–24.99)	86,815 (44.0)	38,025 (47.5)	48,790 (41.6)	48,043 (52.3)	38,772 (36.9)
	Overweight (25.00–29.99)	62,993 (31.9)	25,774 (32.2)	37,219 (31.8)	25,303 (27.5)	37,690 (35.8)
	Obese (≥30.00)	33,029 (16.7)	10,696 (13.4)	22,333 (19.1)	9,650 (10.5)	23,379 (22.2)
Health status	Diseased^¥^	109,045 (55.3)	47,878 (59.8)	61,167 (52.2)	49,566 (53.8)	59,479 (56.6)
	Healthy	88,184 (44.7)	32,141 (40.2)	56,043 (47.8)	42,518 (46.2)	45,666 (43.4)

*BMI values of 159 study participants are missing. Expressed as *n* (%); BMI, Body mass index. ^¥^Diseased includes participants diagnosed with confirmed NCDs (hypertension, diabetes, etc.) as well as intermediate metabolic risk states (pre-hypertension and pre-diabetes) identified during screening.

As depicted in Table 2, the major modifiable risk factors identified were tobacco use (16.7%), alcohol consumption (11.3%), exposure to occupational/environmental pollutants (4.4%), and obesity (16.7%). Tobacco abuse and alcohol consumption were predominantly observed among males (67.4 and 87.9%) and Illiterate participants (75.6 and 67.7%, respectively). Exposure to occupational or environmental pollutants was more common among females (56.2%) and urban residents (88.3%). Obesity peaked in the 40–49 (30.8%) and 50–59 (26.4%) year groups and was more prevalent among females (67.6%).

**Table 2:** Modifiable risk factors associated with non-communicable diseases.

Risk factors		Tobacco abuse (*N* = 32,878, 16.7)	Alcohol consumption (*N* = 22,232, 11.3)	Exposure to occupational/Environmental pollutants (*N* = 8,767, 4.4)	BMI (>30) (*N* = 33,029, 16.7)
		*n* (%)	*n* (%)	*n* (%)	*n* (%)
Age (years)	30–39	6,910 (21.0)	5,021 (22.6)	2,265 (25.8)	8,294 (25.1)
	40–49	8,541 (26.0)	6,093 (27.4)	2,358 (26.9)	10,180 (30.8)
	50–59	7,844 (23.9)	5,495 (24.7)	1,980 (22.6)	8,730 (26.4)
	60–69	6,389 (19.4)	3,947 (17.8)	1,496 (17.1)	4,446 (13.5)
	70 and above	3,194 (9.7)	1,676 (7.5)	668 (7.6)	1,379 (4.2)
Gender	Male	22,171 (67.4)	19,541 (87.9)	3,837 (43.8)	10,696 (32.4)
	Female	10,707 (32.6)	2,691 (12.1)	4,930 (56.2)	22,333 (67.6)
Geographical area	Rural	21,356 (65.0)	12,551 (56.5)	1,028 (11.7)	9,650 (29.2)
	Urban	11,522 (35.0)	9,681 (43.5)	7,739 (88.3)	23,379 (70.8)
Occupation	Homemaker	6,346 (19.3)	1,663 (7.5)	2,150 (24.5)	14,961 (45.3)
	Professional	2,417 (7.4)	1,813 (8.2)	635 (7.2)	2,263 (6.9)
	Manual laborer	13,278 (40.3)	9,502 (42.7)	4,532 (51.8)	8,220 (24.8)
	Others	10,837 (33.0)	9,254 (41.6)	1,450 (16.5)	7,585 (23.0)
Education	Illiterate	24,843 (75.6)	15,052 (67.7)	5,637 (64.3)	20,367 (61.7)
	Junior High School	5,530 (16.8)	4,734 (21.3)	1,923 (21.9)	7,808 (23.6)
	Senior High School	1,144 (3.5)	1,069 (4.8)	514 (5.9)	1,854 (5.6)
	Undergraduate	1,361 (4.1)	1,377 (6.2)	693 (7.9)	2,998 (9.1)

BMI, Body Mass Index.

A family history of HTN (21.5%) and DM (20.1%) were the most frequently reported non-modifiable risk factors, especially among participants aged 40–49 years (32.7 and 32.6%, respectively). The prevalence of all non-modifiable risk factors declined progressively with increasing age. Females reported a higher prevalence of family history for most conditions, including DM (59.8%), HTN (60.6%), obesity (63.4%), cancer (60.5%), and chronic respiratory diseases (CRD) (57.0%). Conversely, males showed slightly higher proportions for heart disease (40.6%) and stroke (42.1%). Homemakers accounted for the largest proportion of family history across most non-modifiable risk factors (35–40%), while professionals consistently reported the lowest prevalence across all conditions (<10%). A similar inverse association with education was observed (Table 3).

**Table 3:** Non-modifiable risk factors associated with non-communicable diseases (family history).

Family history		Heart disease (*N* = 6,945,3.5)	Diabetes (*N* = 39,714, 20.1)	Hypertension (*N* = 42,411, 21.5)	Obesity (*N* = 6,203, 3.1)	Stroke (*N* = 11,506, 5.8)	Cancer (*N* = 7,063, 3.6)	CRD (*N* = 8,336, 4.2)
		*n* (%)	*n* (%)	*n* (%)	*n* (%)	*n* (%)	*n* (%)	*n* (%)
Age (years)	30–39	1,909 (27.5)	12,252 (30.9)	13,708 (32.3)	2,078 (33.5)	2,907 (25.3)	1,705 (24.2)	2,180 (26.2)
	40–49	2,288 (32.9)	12,927 (32.6)	13,856 (32.7)	1,966 (31.7)	3,613 (31.4)	2,334 (33.0)	2,519 (30.2)
	50–59	1,762 (25.4)	9,083 (22.8)	9,480 (22.4)	1,394 (22.5)	3,023 (26.3)	1,788 (25.3)	2,031 (24.4)
	60–69	793 (11.4)	4,302 (10.8)	4,258 (10.0)	600 (9.7)	1,532 (13.3)	957 (13.5)	1,217 (14.6)
	70 and above	193 (2.8)	1,150 (2.9)	1,109 (2.6)	165 (2.7)	431 (3.7)	279 (4.0)	389 (4.7)
Gender	Male	2,819 (40.6)	15,963 (40.2)	16,730 (39.4)	2,273 (36.6)	4,839 (42.1)	2,788 (39.5)	3,586 (43.0)
	Female	4,126 (59.4)	23,751 (59.8)	25,681 (60.6)	3,930 (63.4)	6,667 (57.9)	4,275 (60.5)	4,750 (57.0)
Geographical area	Rural	2,442 (35.2)	13,880 (34.9)	18,318 (43.2)	2,386 (38.5)	4,604 (40.0)	3,032 (42.9)	4,952 (59.4)
	Urban	4,503 (64.8)	25,834 (65.1)	24,093 (56.8)	3,817 (61.5)	6,902 (60.0)	4,031 (57.1)	3,384 (40.6)
Occupation	Homemaker	2,693 (38.8)	15,380 (38.7)	16,723 (39.4)	2,457 (39.6)	4,090 (35.5)	2,797 (39.6)	3,054 (36.6)
	Professional	700 (10.1)	3,865 (9.7)	3,959 (9.3)	586 (9.4)	928 (8.1)	626 (8.9)	598 (7.2)
	Manual laborer	1,781 (25.6)	9,458 (23.8)	9,735 (23.0)	1,474 (23.8)	3,316 (28.8)	1,689 (23.9)	2,316 (27.8)
	Others	1,771 (25.5)	11,011 (27.7)	11,994 (28.3)	1,686 (27.2)	3,172 (27.6)	1,951 (27.6)	2,368 (28.4)
Education	Illiterate	3,310 (47.7)	20,008 (50.4)	22,622 (53.3)	3,146 (50.7)	6,481 (56.3)	3,798 (53.8)	5,306 (63.7)
	Junior High School	2,148 (30.9)	11,219 (28.3)	11,500 (27.1)	1,771 (28.6)	3,061 (26.6)	1,945 (27.5)	1,853 (22.2)
	Senior High School	496 (7.1)	3,026 (7.6)	2,911 (6.9)	495 (8.0)	729 (6.4)	473 (6.7)	378 (4.5)
	Undergraduate	991 (14.3)	5,460 (13.7)	5,376 (12.7)	790 (12.7)	1,235 (10.7)	847 (12.0)	799 (9.6)

CRD, Chronic respiratory diseases.

Of the screened participants, 109,045 (55.3%) were diagnosed with one or more NCDs, with 48.8% newly diagnosed through screening. The most common diagnoses were HTN (39.2%), high-normal/pre-HTN (20.9%), pre-diabetes (9.4%), and diabetes (5.4%). Among them, nearly half of the hypertensive and diabetic cases were newly diagnosed during screening (Table 4). The sociodemographic details of the diagnosed participants are provided in Supplementary Table S1.

**Table 4:** Common non-communicable diseases in the study population with details of already diagnosed (known case) and newly diagnosed patients.

Provisional diagnosis	Total persons diagnosed	Already diagnosed	Newly diagnosed
	*n* (%)	*n* (%)	*n* (%)
High normal (SBP 130–139 and DBP 85–89)/Pre-hypertension	22,809 (20.9)	3,934 (3.6)	18,609 (17.1)
Hypertension	42,787 (39.2)	23,146 (21.3)	19,558 (18.0)
Pre-diabetes	10,259 (9.4)	3,458 (3.2)	6,783 (6.2)
Diabetes	5,931 (5.4)	4,567 (4.2)	1,353 (1.2)
Dyslipidaemia	637 (0.6)	134 (0.1)	503 (0.5)
Chronic respiratory disease	1,541 (1.4)	1,389 (1.3)	152 (0.1)
Coronary artery disease	373 (0.3)	361 (0.3)	12 (0.0)
Stroke	130 (0.1)	123 (0.1)	7 (0.0)
Cancer	51 (0.0)	42 (0.0)	6 (0.0)
Chronic kidney disease	137 (0.1)	94 (0.1)	25 (0.0)
Multi-morbidity	24,390 (22.5)	18,122 (16.7)	6,253 (5.8)
Total*	109,045	55,370*	53,261*

*Information regarding already/new diagnosis status is missing for 414 patients (0.4%). SBP, Systolic blood pressure; DBP, Diastolic blood pressure.

Binary logistic regression analysis (Table 5) showed that the odds of having an NCD increased progressively with age. Females had significantly lower odds of having an NCD than males (AOR = 0.74, 95% CI: 0.72–0.76; *p* < 0.001). Higher education levels were associated with reduced odds of NCDs, showing a graded inverse relationship (AOR for graduates = 0.74, 95% CI: 0.71–0.77; *p* < 0.001). Compared with underweight participants, those who were overweight (AOR = 2.10, 95% CI: 2.02–2.18), or obese (AOR = 2.61, 95% CI: 2.49–2.72) had significantly higher odds of NCDs (*p* < 0.001 for all). Family history of one or more NCDs increased the likelihood of NCD diagnosis by 35% (AOR = 1.35, 95% CI: 1.32–1.38; *p* < 0.001) and the presence of behavioral risk factors associated with 13% higher odds (AOR = 1.13, 95% CI: 1.10–1.15; *p* < 0.001).

**Table 5:** Socio-demographic factors associated with disease status among screened participants.

Variables	Crude odds ratio (COR)	95% CI	*p* value	Adjusted odds ratio (AOR)*	95% CI	*p* value
Age (in years)						
30–39 (Ref.)	–	–	–	–	–	–
40–49	2.10	2.04–2.15	0.0001	2.01	1.96–2.06	0.0001
50–59	3.30	3.22–3.40	0.0001	3.22	3.13–3.30	0.0001
60–69	4.10	3.98–4.22	0.0001	4.30	4.17–4.43	0.0001
≥70	4.61	4.44–4.80	0.0001	5.13	4.92–5.35	0.0001
Gender*						
Male (Ref.)	–	–	–	–	–	–
Female	0.73	0.72–0.75	0.0001	0.74	0.72–0.76	0.0001
Education						
Junior High School (Ref.)	–	–	–	–	–	–
Senior High School	0.90	0.89–0.92	0.0001	0.91	0.88–0.93	0.0001
Undergraduate	0.84	0.80–0.87	0.0001	0.82	0.79–0.86	0.0001
Graduate	0.79	0.76–0.82	0.0001	0.74	0.71–0.77	0.0001
Occupation						
Homemaker (Ref.)	–	–	–	–	–	–
Professional	0.80	0.78–0.82	0.0001	1.09	1.04–1.14	0.0001
Manual Labor	0.85	0.81–0.88	0.0001	0.75	0.73–0.77	0.0001
Others (Businessmen and retired persons)	0.70	0.69–0.72	0.0001	0.95	0.92–0.98	0.001
Residence						
Rural (Ref.)	–	–	–	–	–	–
Urban	1.12	1.10–1.14	0.0001	1.00	0.99–1.02	>0.05
Body mass index						
Underweight (<18.5 kg/m^2^) (Ref.)	–	–	–	–	–	–
Normal weight (between 18.5 and 24.99 kg/m^2^)	1.36	1.31–1.41	0.0001	1.49	1.43–1.55	0.0001
Overweight (25.0–29.9)	1.90	1.83–1.97	0.0001	2.10	2.02–2.18	0.0001
Obese (30.0 and above)	2.27	2.18–2.36	0.0001	2.61	2.49–2.72	0.0001
Family history (heart disease, diabetes, hypertension, obesity, stroke, cancer, chronic respiratory disease, multiple family history)						
No (Ref.)	–	–	–	–	–	–
Yes	1.24	1.22–1.26	0.0001	1.35	1.32–1.38	0.0001
Habits (tobacco abuse, alcohol consumption, exposure to occupational/environmental pollutants, multiple risk factors)						
No (Ref.)	–	–	–	–	–	–
Yes	1.22	1.20–1.24	0.0001	1.13	1.10–1.15	0.0001
Physical activity						
No (Ref.)	–	–	–	–	–	–
Yes	0.68	0.66–0.70	0.0001	1.03	0.99–1.06	>0.05

Dependent variable: Diseased/Healthy (Ref.). *Odd’s ratio adjusted for independent variables: age, gender, education, occupation, residence, body mass index, family history, habits and physical activity.

The mean number of risk factors increased with age up to 40–49 years and declined thereafter (Table 6). Females reported fewer risk factors (0.78 ± 1.01) than males (1.18 ± 1.20), corresponding to a 34% lower odds (AOR = 0.66, 95% CI: 0.65–0.67; *p* < 0.001). Urban residents had a marginally higher mean number of risk factors than rural participants (0.97 ± 1.14 vs. 0.92 ± 1.08; AOR = 1.05, 95% CI: 1.03–1.06; *p* < 0.001). Among occupational groups, professionals had the highest mean risk factor score (1.27 ± 1.23), although their adjusted odds ratio was slightly lower than that of homemakers (AOR = 0.96, 95% CI: 0.93–0.99; *p* = 0.008). Manual laborers showed fewer risk factors (AOR = 0.92, 95% CI: 0.91–0.94; *p* < 0.001). When stratified by diagnosis, individuals with dyslipidemia (1.45 ± 1.31; AOR = 1.70, 95% CI: 1.54–1.89) and multimorbidity (1.10 ± 1.22; AOR = 1.34, 95% CI: 1.31–1.37) had the highest number of risk factors. Participants with DM, HTN, or CRD also exhibited significantly higher adjusted odds (1.17–1.29; *p* < 0.001). Conversely, those with chronic kidney disease (CKD) showed a lower mean number of risk factors (0.55 ± 0.83; AOR = 0.68, 95% CI: 0.51–0.90; *p* = 0.007).

**Table 6:** Mean number of risk factors for non-communicable diseases and independent effects of covariates on risk factors.

Variables		Mean no. of risk factors (mean ± SD)	95% CI	Adjusted odds ratio*	95% CI	*p* value
Age	30–39 (Ref.)	0.99 ± 1.14	0.98–1.00	–	–	–
	40–49	1.10 ± 1.18	0.09–1.11	1.07	1.06–1.09	0.0001
	50–59	0.97 ± 1.13	0.96–0.98	0.95	0.93–0.96	0.0001
	60–69	0.76 ± 0.99	0.75–0.77	0.72	0.71–0.74	0.0001
	≥70	0.60 ± 0.86	0.58–0.61	0.54	0.53–0.56	0.0001
Gender	Male (Ref.)	1.18 ± 1.20	1.17–1.19	–	–	–
	Female	0.78 ± 1.01	0.78–0.79	0.66	0.65–0.67	0.0001
Geographical Area	Rural (Ref.)	0.92 ± 1.08	0.91–0.93	–	–	–
	Urban	0.97 ± 1.14	0.96–0.97	1.05	1.03–1.06	0.0001
Education	Junior High School (Ref.)	0.82 ± 1.04	0.81–0.82	–	–	–
	Senior High School	1.16 ± 1.19	1.15–1.17	1.29	1.27–1.31	0.0001
	Undergraduate	1.25 ± 1.23	1.22–1.28	1.34	1.30–1.38	0.0001
	Graduate	1.37 ± 1.26	1.35–1.40	1.42	1.38–1.45	0.0001
Occupation	Homemaker (Ref.)	0.79 ± 1.03	0.79–0.80	–	–	–
	Professional	1.27 ± 1.23	1.25–1.29	0.96	0.93–0.99	0.008
	Manual laborer	0.92 ± 1.09	0.91–0.93	0.92	0.91–0.94	0.0001
	Others (Businessmen and retired persons)	1.11 ± 1.19	1.0–1.12	1.01	0.99–1.03	0.353
Diagnosis	Healthy (Ref.)	0.86 ± 1.06	0.85–0.87	–	–	–
	High normal (SBP 130–139 and DBP 85–89)/Pre-hypertension	0.91 ± 1.09	0.89–0.92	1.07	1.04–1.09	0.0001
	Hypertension	1.00 ± 1.14	0.99–1.01	1.23	1.21–1.25	0.0001
	Pre-diabetes	1.00 ± 1.12	0.98–1.03	1.17	1.13–1.20	0.0001
	Diabetes	1.12 ± 1.19	1.09–1.15	1.29	1.24–1.34	0.0001
	Dyslipidaemia	1.45 ± 1.31	1.35–1.55	1.70	1.54–1.89	0.0001
	Chronic respiratory disease	1.05 ± 1.19	0.99–1.11	1.25	1.16–1.34	0.0001
	Coronary artery disease	0.99 ± 1.20	0.86–1.11	1.39	1.20–1.60	0.0001
	Stroke	1.08 ± 1.27	0.86–1.30	1.20	0.94–1.53	0.142
	Cancer	0.80 ± 1.06	0.51–1.10	1.12	0.74–1.69	0.600
	Chronic kidney disease	0.55 ± 0.83	0.41–0.70	0.68	0.51–0.90	0.007
	Multimorbidity	1.10 ± 1.22	1.09–1.12	1.34	1.31–1.37	0.0001

Dependent variable: Mean no. of risk factors. *Odds ratio adjusted for independent variables: age, gender, geographical area, education, occupation, diagnosis.

## 4 Discussion

The Integrated NPCDCS program (Homeopathy along with Yoga), through opportunistic screening, aimed to estimate the prevalence of pre-defined NCDs in tandem with the NPCDCS program of Government of India (21). Since its launch in 2010 and the adoption of the National NCD Monitoring framework (12), many studies were conducted to assess the prevalence of risk factors and NCDs at national, state and district levels. NCD risk factor surveillance through the STEP approach is recommended by WHO (15), and by adapting some of its approaches, the present study screened a large number of participants from four districts in different states of India through LSD clinics and outreach health camps. This large-scale analysis of screened population under the integrated NPCDCS pilot project provides an extensive overview of the distribution and determinants of NCDs and their associated risk factors. Nearly 55.3% of individuals had at least one NCD, indicating the substantial burden of chronic diseases in the community. The predominance of HTN (39.2%) and DM (14.8%), alongside high rates of overweight (31.9%) and obesity (16.7%), aligns with global and national evidence on the epidemiological transition toward NCD dominance (1, 4, 8, 22). A considerable proportion of participants exhibited intermediate metabolic conditions, namely pre-hypertension and pre-diabetes. Although not classified as established NCDs, these conditions are important early markers of cardiometabolic risk and key targets for preventive interventions in population-based screening programs.

The overall prevalence of NCDs observed in this study aligns with findings from the National NCD Monitoring Survey (NNMS), which reported that HTN and DM are the most common NCDs among Indian adults (23). Nearly half of the hypertensive and diabetic cases in this study were newly diagnosed through opportunistic screening, highlighting a critical gap in early detection—similar to observations from the ICMR-INDIAB study and other community-based surveys (24, 25). These findings reinforce the need for active surveillance and community-level screening, as recommended in the WHO STEPwise approach (15) and India’s National Action Plan for NCD Prevention and Control (13). The observed pattern of higher NCD prevalence among older adults is consistent with previous studies (3, 25–27). The fivefold increase in adjusted odds among those aged over 70 years supports the global understanding of age as a dominant, non-modifiable risk factor for chronic disease (10, 28). However, the decline in the mean number of risk factors in the oldest age groups, as seen here, may reflect survivorship bias or selective mortality, as also noted by Chauhan et al. (3) in older Indian populations.

Gender differences in NCD burden were notable, with females showing lower odds of NCDs compared to males. This finding corroborates the NNMS and Punjab STEPS surveys (23, 29), which reported higher behavioral risk exposure among men, particularly tobacco and alcohol use. However, the higher prevalence of obesity among women, as seen in this study, aligns with previous findings from Kerala, Haryana, and Tamil Nadu (26, 30, 31), and may be linked to sociocultural patterns of physical inactivity and dietary practices among middle-aged homemakers. Educational attainment was inversely associated with NCD risk, echoing evidence from the Longitudinal Aging Study in India (LASI) and other national datasets (3, 8). While education often confers protective effects through enhanced health literacy and access to preventive services, our finding that graduates exhibited more behavioral risk factors may reflect lifestyle-related exposures linked to urbanization and sedentary work environments (4, 32). The complex interplay between education, occupation, and lifestyle underscores the dual burden faced by India’s transitioning middle class (5–7).

Urban participants showed higher prevalence of overweight, obesity, and metabolic risk factors, in line with prior studies across India and South Asia (26, 29, 32–34). Although residence lost significance in the adjusted model, urban lifestyles—characterized by reduced physical activity, higher caloric intake, and environmental pollution—remain crucial contributors to NCD risk (31, 32). Manual laborers demonstrated lower odds of NCDs, consistent with their higher levels of occupational physical activity (27, 30), whereas professionals exhibited marginally higher odds, reflecting the sedentary occupational hazard noted in earlier urban surveys (29, 35). Behavioral risk factors were widespread, with tobacco use (16.7%) and alcohol consumption (11.3%) being predominant—figures comparable to national estimates from GATS-2 (36) and the NNMS (23). The tobacco consumption in India has decreased from 34.6% reported by GATS 1 (2009–10) (37) to 28.6% reported by GATS 2 (2016–17) (36). In 2020, 22.3% of the world’s population consumed tobacco in some form, and it kills up to half of its long-term users (38). The data showed that despite their best efforts, countries have a long way to go in curbing the menace of tobacco use. The strong male and rural preponderance of these habits mirror national trends (37, 38). The modest association between combined behavioral risk exposure and NCD occurrence (AOR = 1.13) indicates cumulative, yet modifiable, risk potential.

Obesity emerged as a powerful predictor of NCDs, with more than twofold higher odds, supporting previous national analyses (27, 39). This aligns with WHO’s recognition of overweight and obesity as key global NCD drivers (1, 28). The coexistence of obesity and hypertension/diabetes in this cohort mirrors findings from CURES and ICMR studies (34, 40), reinforcing the clustering of metabolic risk in middle-aged Indian adults. The strong association between family history and NCDs (AOR = 1.35) further corroborates the intergenerational risk documented in previous Indian and regional surveys (41, 42).

The findings highlight both the magnitude of undiagnosed NCDs and the persistence of modifiable risk factors, suggesting gaps in primary prevention and early detection. Strengthening community-based screening under NPCDCS (12), integrating health promotion with existing PHC systems (7), and addressing urban lifestyle risk through multisectoral interventions are essential. The gendered and occupational variations identified call for tailored strategies—particularly for homemakers and low-literacy populations—to improve behavioral change communication. Given the WHO’s call for accelerated NCD control (10), this study highlights the utility of large-scale screening data for policy formulation and monitoring progress toward India’s national NCD targets (13). Reducing risk factor clustering through targeted interventions can contribute substantially to achieving Sustainable Development Goal (SDG) 3.4, which aims to reduce premature NCD mortality by one-third by 2030 (10). Future cross-sectional studies may explore the relationship between conventional risk factors for NCDs and metabolic profiling of patients, including emerging biomarkers (including hs-CRP, insulin resistance markers, adipokines, and apolipoproteins) and related cardiometabolic indicators. Such studies may help better understand potential associations between metabolic markers and individual risk profiles, thereby strengthening risk stratification and preventive strategies in population-based screening programs.

## 5 Strengths and limitations

This is the first-of-its-kind study in which the prevalence of NCDs and their risk factors were assessed in an ALDCs under the NPCDCS program. The study has presented robust estimates from four distinct geographical locations of India. The major strengths of this study lie in its large sample size, real-world implementation under a national program, and comprehensive assessment of both modifiable and non-modifiable determinants. However, in the cross-sectional design, potential reporting bias in self-reported behaviors cannot be ruled out. The difference in prevalence estimates between this study and similar studies may be due to differences in methodology, including study objectives, study design, questionnaires, target population, and sampling strategy. There might be under- or over-reporting of risk factors like tobacco and alcohol consumption due to social desirability bias. Despite these limitations, the study provides valuable insights into the evolving epidemiology of NCDs in India and offers empirical evidence to inform region-specific prevention strategies.

## 6 Conclusion

This large-scale screening under the Integrated NPCDCS program (Homeopathy along with yoga) highlights a substantial burden of undiagnosed NCDs and a high prevalence of both modifiable and non-modifiable risk factors among Indian adults. Almost half of the cases were newly diagnosed, emphasizing the importance of opportunistic screening. Tailored, context-specific prevention programs through an integrated approach focusing on lifestyle modification and enhanced health services in India may help address the growing NCD burden.

## Data Availability

The original contributions presented in the study are included in the article/Supplementary material, further inquiries can be directed to the corresponding author.

## Group members of NPCDCS-AYUSH integration project study group

Krishna: Avanigadda- Dr. P. Bhaskar, Dr. S. Lokesh Kumar; Challapalli- Dr. I. Lakshmi Deepak, Dr. G. Swathi; Gudivada- Dr. Ch. Aruna Sri, Dr. K. Rajesh; Machilipatnam- Dr. M. Jaya Lakshmi, Dr. S. Siva Kumar; Nandigama- Dr. M. Jaya Subhashini, Dr. V. Pragathi Pawan; Nuziveedu- Dr. N. Sree Hanumantha Rao, Dr. R. Jyothsna; Tiruvuru- Dr. K. Kishore, Dr. Masarath Sulthana; Vissannapeta- Dr. B. Poorna Chandra Rao; Vuyyuru- Dr. K. Srinivasulu, Dr. Y. Surya Narayana; Darjeeling: Kalimpong- Dr. Pulakendu Bhattacharya, Dr. Baishakhi Ghosh, Dr. Ruma Debbarma; Siliguri (CRU)-Dr Abhiram Banerjee; Phansidewa-Dr Indra Dev Pratap; Bijanbari-Dr Santosh Kumar Suman; Mirik- Dr. Sabiha Rafique, Dr. Chandranath Ghosh, Dr. Souvik Jana; Pedong- Dr. Md. Mostafijur Rahaman, Dr. Nicky T. Bhutia; Sambalpur: OMCH&R-Dr. Pankaj Shukla; Themra- Dr. Mahendra Agrahari; Jujumura- Dr. Sanjay Debnath, Dr. Debashish Sharma Sarkar; Nashik: Dr. Nitesh Jangid; Igatpuri- Dr. Xinix Xavier, Dr. Yogini Jadhav, Kalwan: Dr. Prajakta Dhanvijay.
