# Supplementary material for: Clinico-epidemiological profile findings of the screened population under NPCDCS–Ayush (Integration of Homeopathy along with Yoga): a pilot project
Source: Front Public Health. 2026 May 21;14:1793635. doi: 10.3389/fpubh.2026.1793635 (PMC13233674; doi:10.3389/fpubh.2026.1793635)
Supplement: Supplementary file 1 [file Data_Sheet_1.pdf]

Name of the District………………………………………………

Name of the CHC/BPHC………………………………………………

Screening No……. **Date: ………………**

A. **Screening Level:** ☐ At LSD Clinic at identified CHC (1) ☐ At Out-reach camp (2)

B. GENERAL INFORMATION

**State_____________**	**District___________**	**Block ________**	**Sub center_________**
**Name:**<br>**Age (in years):**<br>**Gender**<br>☐ Male (1)<br>☐ Female (2)<br>**Aadhar number:**	**Address**	**Ph. / Mobile / Neighborhood Mobile**	**Name of Father/Mother/ Husband/ Wife/ Guardian**
**Occupation**<br>☐ House wife (1)<br>☐ Professional (2)<br>☐ Manual Laborer (3)<br>☐ Others Specify (4) …………….		**Education**<br>☐ Junior high school or less (1)<br>☐ Senior high school level (2)<br>☐ Undergraduate level (3)<br>☐ Graduate level (4)	

C. ANTHROPOMETRIC INFORMATION:

1. **Height (in mt) …….** 2. **Weight (in Kg) ………… 3. BMI_____**<br>4. **Waist circumference (WC): _ _ _ _ _ _ cm**<br>5. **Hip circumference (HC): _ _ _ _ _ cm**	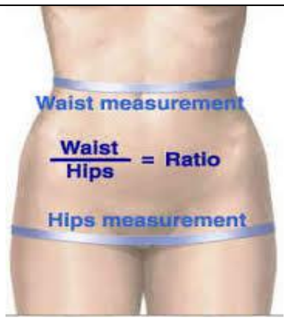

D. FAMILY HISTORY

1. Heart disease	☐ Yes (1)	☐ No (2)
2. Diabetes	☐ Yes (1)	☐ No (2)
3. Hypertension	☐ Yes (1)	☐ No (2)
4. Obesity	☐ Yes (1)	☐ No (2)
5. Stroke	☐ Yes (1)	☐ No (2)
6. Cancer	☐ Yes (1)	☐ No (2)
7. Chronic respiratory diseases (COPD & Asthma)	☐ Yes (1)	☐ No (2)

8. Multiple family history

(of above diseases) ☐ Yes (1) ☐ No (2)

If yes, mention the number from the above list…………………

E. HABITS

Do you smoke or consume smokeless products such as Gutkha or Khaini?	☐ Yes (1)	☐ No (2)
	If Yes ☐ Current or	☐ History
Alcohol consumption	☐ Yes (1)	☐ No (2)
	If Yes ☐ Current or	☐ History
Exposure to occupational /environmental pollutants related to CRD	☐ Yes (1)	☐ No (2)
(If yes, specify the pollutant ________________)		
If multiple habits, please specify…………………………		

F. RISK ASSESSMENT FOR LIFE STYLE DISORDERS

Indian Diabetes Risk Score (IDRS)

**Categorized Risk factors**	**Score**	**Score of the patient**
**Age**		
< 35 years	0	
35 to 49 years	20	
≥50 years	30	
**Abdominal obesity**		
Waist circumference females <80cm &males < 90cm	0	
Female 80-89cm, Male 90-99cm	10	
Female ≥90cm, Male ≥100cm	20	
**Physical activity**		
Vigorous exercise or strenuous at work	0	

Categorized Risk factors	Score	Score of the patient
Moderate exercise at work/home	10	
Mild exercise at work/home	20	
No exercise and sedentary at work/home	30	
**Family history**		
Two non-diabetic parents	0	
Either parent diabetic	10	
Both parents diabetic	20	
Maximum score	100	

Score ≥60: High risk, 30-50: Medium risk, <30: Low risk.

SCORE OF THE PATIENT: …………………

G. INVESTIGATIONS/ PHYSICAL EXAMINATION

1. Random Blood Sugar level……………mg /dl

☐ Non-diabetic/Normal (<140 mg/dl) (1) ☐ Abnormal (≥140 mg/dl) (2)

If Abnormal:

☐ Pre-diabetic (≥140mg/dl- <200 mg/dl) (1) ☐ Diabetic (≥200 mg/dl) (2)

2. Blood pressure Systolic……………mm Hg **Diastolic ……………** mm Hg

- Reading 1 SBP…………………… DBP……………………
- Reading 2 SBP…………………… DBP……………………
- Reading 3 SBP…………………… DBP……………………
- Average SBP…………………… DBP……………………

☐ Normal (<120/<80) (1) ☐ Pre-hypertension (120-139/80-89) (2)

☐ HTN Stage 1 (140-159/90-99) (3) ☐ HTN Stage 2 (≥160/ ≥100) (4)

H. Early detection: Ask if patient has any of these Symptoms CRD/Cancer

Women and Men	Yes (1)/ No (2)	Women only	Yes (1)/No (2)
Shortness of breath		Lump in the breast	
Coughing more than 2 weeks		Blood stained discharge from the nipple	
Blood in sputum		Change in shape and size of breast	
Difficulty in opening mouth		Bleeding between periods	
Ulcers/patch/growth in the mouth that has not healed in two weeks		Bleeding after menopause/ intercourse	
Any change in the tone of voice		Foul smelling vaginal discharge	
Unexplained weight loss			

**Women and Men**	**Yes (1)/ No (2)**	**Women only**	**Yes (1)/No (2)**
Chronic change in bowel or bladder habits			
Chronic indigestion or difficulty in swallowing			
Unusual bleeding or discharge			
Obvious change in the size, color, shape, or thickness of a wart or mole			

I. **Whether the person was screened earlier** ☐ Yes (1) ☐ No (2)

If yes, mention the previous screening number………………………

J. **If yes, whether the person was healthier earlier** ☐ Yes (1) ☐ No (2)

If yes, is the person, currently ☐ Healthier (1) ☐ Diseased (NCDs) (2)

If, diseased, (Mention the provisional diagnosis) ………………

K. HEALTH STATUS OF THE PERSON: ☐ **Healthy/Normal (1)** ☐ **Diseased (2)**

L. DIAGNOSIS : ☐ **Already Diagnosed (1)** ☐ **Newly Diagnosed (2)**

M. PROVISIONAL DIAGNOSIS

☐ **Pre-Hypertension (1)** ☐ **HTN Stage – I (2)** ☐ **HTN Stage-II (3)**

☐ **Pre-Diabetes (4)** ☐ **Diabetes (5)** ☐ **Dyslipidemia (6)**

☐ **CRD (7)** ☐ **CAD (8)** ☐ **Stroke (9)**

☐ **Cancer (10)** ☐ **Multi-Morbidity (11)**

If Multi-Morbidity, mention different disease conditions from the identified NCDs ……………………………………

If the person is suffering from any NCD other than the identified NCDs, mention the disease………………………

O. Whether the case is documented ☐ Yes (1) ☐ No (2)

F. In case of any abnormal findings from above refer to:

(Name of CHC / District NCD Clinic / Any other)

**Name & signature**<br>**(Homoeopathic doctor)**	**Signature**<br>**(Multitask Health Worker)**
