# Supplementary material for: Clinico-epidemiological profile findings of the screened population under NPCDCS–Ayush (Integration of Homeopathy along with Yoga): a pilot project
Source: Front Public Health. 2026 May 21;14:1793635. doi: 10.3389/fpubh.2026.1793635 (PMC13233674; doi:10.3389/fpubh.2026.1793635)
Supplement: Supplementary file 2 [file Table_1.docx]

**Supplementary Table 1: Socio-demographic details of the Diagnosed population (N=109045)**

|  | | **High Normal Blood Pressure** | **HTN** | **Pre DM** | **DM** | **Dyslipidemia** | **CRD** | **CAD** | **Stroke** | **Cancer** | **CKD** | **MM** |
| --- | --- | --- | --- | --- | --- | --- | --- | --- | --- | --- | --- | --- |
|  | | n (%) | n (%) | n (%) | n (%) | n (%) | n (%) | n (%) | n (%) | n (%) | n (%) | n (%) |
| **Age(years)** | **30-39** | 5482(24.0) | 6598(15.4) | 2111(20.6) | 961(16.2) | 258(40.5) | 380(24.7) | 24(6.4) | 14(10.8) | 5(9.8) | 7(5.1) | 2332(9.6) |
|  | **40-49** | 6108(26.8) | 10616(24.8) | 2872(28.0) | 1645(2.7) | 213(33.4) | 364(23.6) | 38(10.2) | 31(23.8) | 9(17.6) | 30(21.9) | 5593(22.9) |
|  | **50-59** | 5333(23.4) | 11401(26.6) | 2653(25.8) | 1762(29.7) | 129(20.3) | 353(22.9) | 63(16.9) | 38(29.2) | 19(37.3) | 49(35.8) | 7236(29.7) |
|  | **60-69** | 3960(17.4) | 9414(22.1) | 1838(17.9) | 1162(19.6) | 29(4.6) | 283(18.4) | 100(26.8) | 30(23.1) | 12(23.5) | 39(28.5) | 6318(25.9) |
|  | **70 and above** | 1926(8.4) | 4758(11.1) | 785(7.7) | 401(6.8) | 8(1.3) | 161(10.4) | 148(39.7) | 17(13.1) | 6(11.8) | 12(8.7) | 2911(11.9) |
| **Gender** | **Male** | 9612(42.1) | 18161(42.4) | 4497(43.8) | 2770(46.7) | 168(26.4) | 694(45.0) | 218(58.4) | 87(66.9) | 12(23.5) | 87(63.5) | 11572(47.4) |
|  | **Female** | 13197(57.9) | 24626(57.6) | 5762(56.2) | 3161(53.3) | 469(73.6) | 847(55.0) | 155(41.6) | 43(33.1) | 39(76.5) | 50(36.5) | 12818(52.6) |
| **Geographical area** | **Rural** | 10536(46.2) | 21332(49.9) | 4600(44.8) | 2249(37.9) | 143(22.4) | 582(37.8) | 111(29.8) | 37(28.5) | 15(29.4) | 3(2.2) | 9958(40.8) |
|  | **Urban** | 12273(53.8) | 21455(50.1) | 5659(55.2) | 3682(62.1) | 494(77.6) | 959(62.2) | 262(70.2) | 93(71.5) | 36(70.6) | 134 (97.8) | 14432(59.2) |
| **Occupation** | **Homemaker** | 8209(36.0) | 16218(37.9) | 3667(35.8) | 2009(33.8) | 245(38.5) | 501(32.6) | 99(26.5) | 24(18.5) | 30(58.8) | 25(18.2) | 8628(35.4) |
|  | **Professional** | 1514(6.6) | 2603(6.1) | 785(7.7) | 426(7.2) | 29(4.6) | 91(5.9) | 21(5.6) | 4(3.1) | 1(2.0) | 1(0.7) | 1683(6.9) |
|  | **Manual Labourer** | 7208(31.6) | 12130(28.4) | 3046(29.7) | 1831(30.9) | 249(39.1) | 572(37.2) | 135(36.2) | 51(39.2) | 11(21.6) | 50(36.5) | 6714(27.5) |
|  | **Others** | 5857(25.7) | 11820(27.6) | 2750(26.8) | 1657(28.0) | 114(17.9) | 372(24.2) | 118(31.6) | 51(39.2) | 9(17.6) | 61(44.5) | 7352(30.2) |
| **Education** | **Illiterate** | 15503(68.0) | 31148(72.8) | 6808(66.4) | 3909(66.0) | 429(67.4) | 995(64.8) | 291(78.0) | 94(72.3) | 40(78.4) | 133(97.1) | 16777(68.8) |
|  | **Junior High School** | 4540(19.9) | 7710(18.0) | 2150(21.0) | 1302(22.0) | 130(20.4) | 327(21.3) | 47(12.6) | 24(18.5) | 9(17.6) | 3(2.2) | 4966(20.4) |
|  | **Senior High School** | 1175(5.2) | 1535(3.6) | 500(4.9) | 284(4.8) | 25(3.9) | 82(5.3) | 16(4.3) | 4(3.1) | 1(2.0) | 0(0.0) | 1050(4.3) |
|  | **Undergraduate** | 1570(6.9) | 2377(5.6) | 790(7.7) | 428(7.2) | 53(8.3) | 132(8.6) | 19(5.1) | 8(6.2) | 1(2.0) | 1(0.7) | 1584(6.5) |
| **Overall** |  | **22809 (20.9)** | **42787(39.2)** | **10259(9.4)** | **5931(5.4)** | **637(0.6)** | **1541(1.4)** | **373(0.3)** | **130(0.1)** | **51(0.0)** | **137(0.1)** | **24390(22.5)** |
